# New Genes Involved in Mild Stress Response Identified by Transposon Mutagenesis in *Lactobacillus paracasei*

**DOI:** 10.3389/fmicb.2018.00535

**Published:** 2018-03-23

**Authors:** Aurore Palud, Hélène Scornec, Jean-François Cavin, Hélène Licandro

**Affiliations:** Université de Bourgogne Franche-Comté, AgroSup Dijon, PAM UMR A 02.102, Dijon, France

**Keywords:** lactic acid bacteria, transposon mutants, mild stresses, stress response genes, bacterial adaptation

## Abstract

Lactic acid bacteria (LAB) are associated with various plant, animal, and human niches and are also present in many fermented foods and beverages. Thus, they are subjected to several stress conditions and have developed advanced response mechanisms to resist, adapt, and grow. This work aimed to identify the genes involved in some stress adaptation mechanisms in LAB. For this purpose, global reverse genetics was applied by screening a library of 1287 *Lactobacillus paracasei* transposon mutants for mild monofactorial stresses. This library was submitted independently to heat (52°C, 30 min), ethanol (170 g.L^−1^, 30 min), salt (NaCl 0.8 M, 24 h), acid (pH 4.5, 24 h), and oxidative (2 mM H_2_O_2_, 24 h) perturbations which trigger mild monofactorial stresses compatible with bacterial adaptation. Stress sensitivity of mutants was determined either by evaluating viability using propidium iodide (PI) staining, or by following growth inhibition through turbidity measurement. The screening for heat and ethanol stresses lead respectively to the identification of 63 and 27 genes/putative promoters whose disruption lead to an increased sensitivity. Among them, 14 genes or putative promoters were common for both stresses. For salt, acid and oxidative stresses, respectively 8, 6, and 9 genes or putative promoters were identified as essential for adaptation to these unfavorable conditions, with only three genes common to at least two stresses. Then, RT-qPCR was performed on selected stress response genes identified by mutant screenings in order to evaluate if their expression was modified in response to stresses in the parental strain. Eleven genes (membrane, transposase, chaperone, nucleotide and carbohydrate metabolism, and hypothetical protein genes) were upregulated during stress adaptation for at least two stresses. Seven genes, encoding membrane functions, were upregulated in response to a specific stress and thus could represent potential transcriptomic biomarkers. The results highlights that most of the genes identified by global reverse genetics are specifically required in response to one stress and that they are not differentially transcribed during stress in the parental strain. Most of these genes have not been characterized as stress response genes and provide new insights into the adaptation of lactic acid bacteria to their environment.

## Introduction

*Lactobacillus casei/paracasei* is one of the most emblematic groups of lactic acid bacteria (LAB), probably because it was one of the first studied and marketed for its benefits on health in the form of dairy products (Saxelin et al., 2005). Besides, strains of this group are nonstarter LAB in ripened cheeses, where they contribute to flavor development. Comparative genomics demonstrated that this versatile group is highly adaptable to various niches, thus it can be present and active in plethora of environments such as dairy and plant products, oral, intestinal, and reproductive tracts of humans and animals (Cai et al., 2010; Douillard et al., 2013; Duar et al., 2017). Moreover, among *Lactobacillus* genus, *L. casei, paracasei*, and *rhamnosus* species are widely used and studied for their probiotic function. For example, it was demonstrated that some strains can inhibit pathogenic bacteria (Ingrassia et al., 2005) and prevent diarrhea (de Roos and Katan, 2000). Considering the importance of these strains for industrial application as well as for health effects, many publications focused on stress response in *L. casei/paracasei* group, in particular when they are exposed to acid (Broadbent et al., 2010), cold (Beaufils et al., 2007), and bile stresses (Wu et al., 2010; Alcantara and Zuniga, 2012; Hamon et al., 2012). These data are discussed by Hosseini Nezhad et al. (2015).

Although LAB are fastidious because they possess numerous auxotrophies, and that only a few strains are genetically manipulable, many studies have been dedicated to the comprehension of the high adaptability of LAB to environmental stresses, guided by fundamental issues and potential industrial applications. Physiological approaches have highlighted the phenomenon of stress adaptation, i.e., the transient improvement of stress resistance when LAB are submitted to a first stress of mild intensity (Andrade-Linares et al., 2016). This phenomenon, also called priming stress, is a key factor for the development of active starters or probiotics and for their preservation. Then, as transcriptomics and proteomics have progressed, genetic determinants of stress response have been identified (De Angelis and Gobbetti, 2004). Some genes were qualified as “general” stress response genes whereas others were stress-specific. Some of the most studied proteins are Hsp (Heat shock protein; Sugimoto et al., 2008), UspA (Universal stress protein; Kvint et al., 2003; Gury et al., 2009), and those of the ATR (Acid Tolerant Response; Cotter and Hill, 2003). In addition, some genes can be functionally determinant for stress adaptation without, however, exhibiting transcriptomic or proteomic changes. Indeed, comparative genome analyses demonstrated that genes for niche adaptation are mainly carried by plasmids and adaptation islands on the chromosome (Cai et al., 2010; Smokvina et al., 2013). Also, targeted inactivation of genes (allowing reverse genetics) brought new information on LAB stress response. All knowledges generated by these studies were reviewed in a comprehensive and detailed manner by van de Guchte et al. (2002) and Papadimitriou et al. (2015, 2016).

More global approaches such as random mutagenesis or gene expression library have made possible global reverse genetics but they have been only slightly used so far, probably because of the difficulty to develop them. The main reason is that random mutagenesis approaches need the building of a transposon mutant library and then the screening of each mutant impaired for the function of interest in order to identify the corresponding gene(s) responsible for the phenotype. This requires transposon vectors tailored for the bacterial species and is often a limiting factor for LAB which can be difficult to transform, and for which the selective antibiotics panel is limited. To overcome this limitation, the P_junc_-TpaseIS_1223_ system has been developed specifically for *Lactobacillus* genus (Licandro-Seraut et al., 2012). We succeeded in this approach by applying transposon mutagenesis in the frame of the adaptation of *Lactobacillus pentosus* to olive brine, which is a multifactorial stress, and identified five brine sensitive mutants (Perpetuini et al., 2013, 2016). We have demonstrated the strength of this approach by uncovering many new genetic determinants during the early stage of *L. paracasei* establishment at the intestinal level, when bacteria have to cope with numerous changes of their environment (Licandro-Seraut et al., 2014). For this study, thanks to the sequencing of each transposon target of the mutant library, we have set up a non-redundant library of 1,110 transposon mutants in which the transposon target was identified in a coding region for each mutant (Scornec et al., 2014).

In the present work, we revisit the issue of LAB stress response by a global reverse genetics approach. We took advantage of the annotated random transposon library of *L. paracasei* cited above. In order to have a fine resolution, we have chosen to apply simple (monofactorial) stress of mild intensities, compatible with an adaptation of the LAB population. We defined this type of perturbation as “mild stress.” For this purpose, we have implemented suitable screening strategies to study separately the responses to five of the major components that can change when *L. paracasei* is under multifactorial environmental stresses: heat, salt, oxidative, ethanol, and acidic stresses. The cross-checking of the screening results and the transcriptional analysis of selected genes allowed us to identify new genetic determinants of stress response and to qualify them for a “general stress” or “specific stress” response.

## Materials and methods

### Strains and growth conditions

Wild-type (WT) *L. paracasei* ATCC 334 (CIP 107868, Institut Pasteur Collection; Judicial Commission of the International Committee on Systematics of Bacteria, 2008) and its corresponding mutants obtained by random transposon mutagenesis (Licandro-Seraut et al., 2014) were grown statically at 37°C in MRS (Difco). The antibiotic used was 5 μg.mL^−1^ erythromycin (Erm) during mutant preculture. The mutants corresponded to the 1,100 genic mutants already described (Licandro-Seraut et al., 2012) and to 177 new mutants selected among the 8,053 previously annotated mutants (Scornec et al., 2014) because their transposon was inserted at <100 bp upstream of a gene for which no genic mutant had been found. For each mutant, the putative inactivated function was assigned thanks to the genome annotation of this strain (Cai et al., 2010).

### Determination of stress intensities based on WT viability

WT *L. paracasei* was grown until stationary phase because it was the most convenient for the mutant screening. The culture (1 ml) was centrifuged (4,000 g, 4 min, 25°C) and the pellet was suspended with one volume of buffer modified for the stress. Phosphate buffer (pH 6.5) was supplemented with 1 M to 4 M NaCl for salt stress, with 450 μM to 350 mM H_2_O_2_ for oxidative stress and with 150–200 g.L^−1^ ethanol for ethanol stress. For acid stress, the buffer was a 0.2 M KCl/0.2 M HCl solution with pH ranging from 2.0 to 2.4. Exposure to heat stress (48–60°C) was performed in phosphate buffer in a C1000™ Thermal Cycler (Biorad). WT viability after a 30-min exposure to stress was determined by plate counts (three biological replicates).

### Viability screening by propidium iodide

Mutant cultures in stationary phase (20 μl) were put in a white 96-well PCR plate (Biorad), centrifuged, pellets were suspended in 100 μl of stress buffer. Phosphate buffer was supplemented with 170 g.L^−1^ ethanol for ethanol stress. Exposure to heat stress (52°C) was performed in prewarmed phosphate buffer and in a C1000™ Thermal Cycler (Biorad). After a 30-min incubation, bacterial pellets were suspended in 100 μl of 20 μM propidium iodide (PI, Sigma-Aldrich). All centrifugations were done at 4,000 g for 10 min and all suspensions were performed by vortexing (1 min at 1,550 rpm in a Mix mate, Eppendorf). Fluorescence (λ_em_/λ_exc_: 490/635 nm) was measured in a plate reader (Beckman Coulter) to calculate the viability percent with respect to a calibration range of known proportions of “dead” cells of *L. paracasei* WT (heat inactivated, 30 min at 80°C). Two biological replicates were made. When necessary, a third biological replicate was made to confirm the phenotype. Mutants were qualified as sensitive if their viability was less or equal to the WT viability minus twice the standard deviation for the two biological replicates. Corresponding genes were aligned using BLAST (https://blast.ncbi.nlm.nih.gov/Blast.cgi) against all bacteria to determine their specificity.

### Growth inhibition screening by turbidimetry

Mutant cultures in stationary phase were inoculated at 1/20 (v/v) (OD_600 nm_ ≈ 0.2) in 200 μl of modified MRS as specified hereafter. MRS was supplemented with 0.8M NaCl for salt stress, 2 mM H_2_O_2_ for oxidative stress and acidified with HCl until pH 4.5 for acid stress. Microplates were incubated 24 h at 37°C and vortexed (1,000 rpm 1 min in a Mix Mate, Eppendorf) to read the final OD_600 nm_ in a plate reader (Beckman Coulter). Mutants were qualified as sensitive when OD_600 nm_ (24 h) was 1.5-fold lower than that of the WT final OD_600 nm_ (two biological replicates). Corresponding genes were aligned using BLAST against all bacteria to determine their specificity. An alternative method was also used for selected sensitive mutants. After a 30-min stress in modified MRS, bacterial pellets were suspended in MRS to follow the growth at 37°C during 7 h. Mutants were considered sensitive when their growth percentage at 7 h in stress condition compared to the control condition, was inferior to the WT growth percentage minus the value of two standard derivations (two biological repeats).

### Growth inhibition determined by diffusion test assay for oxidative stress

Mutant cultures in stationary phase of growth were inoculated at 1/20 in 20 mL of MRS agar before solidification in a Petri dish. Disks impregnated with 10 μl of 3.5 M H_2_O_2_ were placed on MRS agar. After a 24-hincubation at 37°C, inhibition diameters were measured and mutants were considered as sensitive when their inhibition diameters were significantly higher than that of the WT (Student test, *p* < 0.05, six biological replicates).

### RT-qPCR

For transcriptomic analysis, cells in mid-exponential growth phase (OD_600_ between 0.6 and 0.8) were centrifuged and cell pellets were suspended with one volume of the dedicated media. Thereby, in function of the applied stress, MRS was supplemented with 1 mM H_2_O_2_, 1 M NaCl, 150 g.L^−1^ ethanol, or acidified with HCl to pH 3.0. Phosphate buffer was maintained in a water bath at 50°C for heat stress (four biological replicates). Total RNA isolation, cDNA synthesis, and qPCR was made as previously described (Licandro-Seraut et al., 2008) using TRI Reagent (Sigma Aldrich), DNase I (Roche), iScript™ Reverse Transcription Supermix (Bio-Rad), and SsoAdvanced™ Universal SYBR® Green Supermix (Bio-Rad). Primers were designed by using Primer3Plus (Untergasser et al., 2007) (Table S1). Quantitative PCR (qPCR) were performed using a CFX96 Touch™ Real-Time PCR Detection System (Bio-Rad) in triplicate, in a 20 μl-reaction mixture. Cq (threshold value) calculation was determined by a regression model of the CFX Manager™ Software. Gene expression was calculated using 2^−ΔΔCT^ method (Livak and Schmittgen, 2001). In order to select appropriate reference genes, 10 housekeeping genes (*fusA, ileS, lepA, leuS, mutL, pcrA, pyrG, recA, recG*, and *rpoB*) were tested with all experimental conditions and analyzed using the CFX Manager™ Software. The genes *recG* and *rpoB* were selected as the references because they displayed the lowest *M*-values (0.57) and coefficients of variation (0.20), meaning that they have the most stable expression in the tested stress conditions.

## Results

### Development of the mutant screening strategy

**Figure 1:**
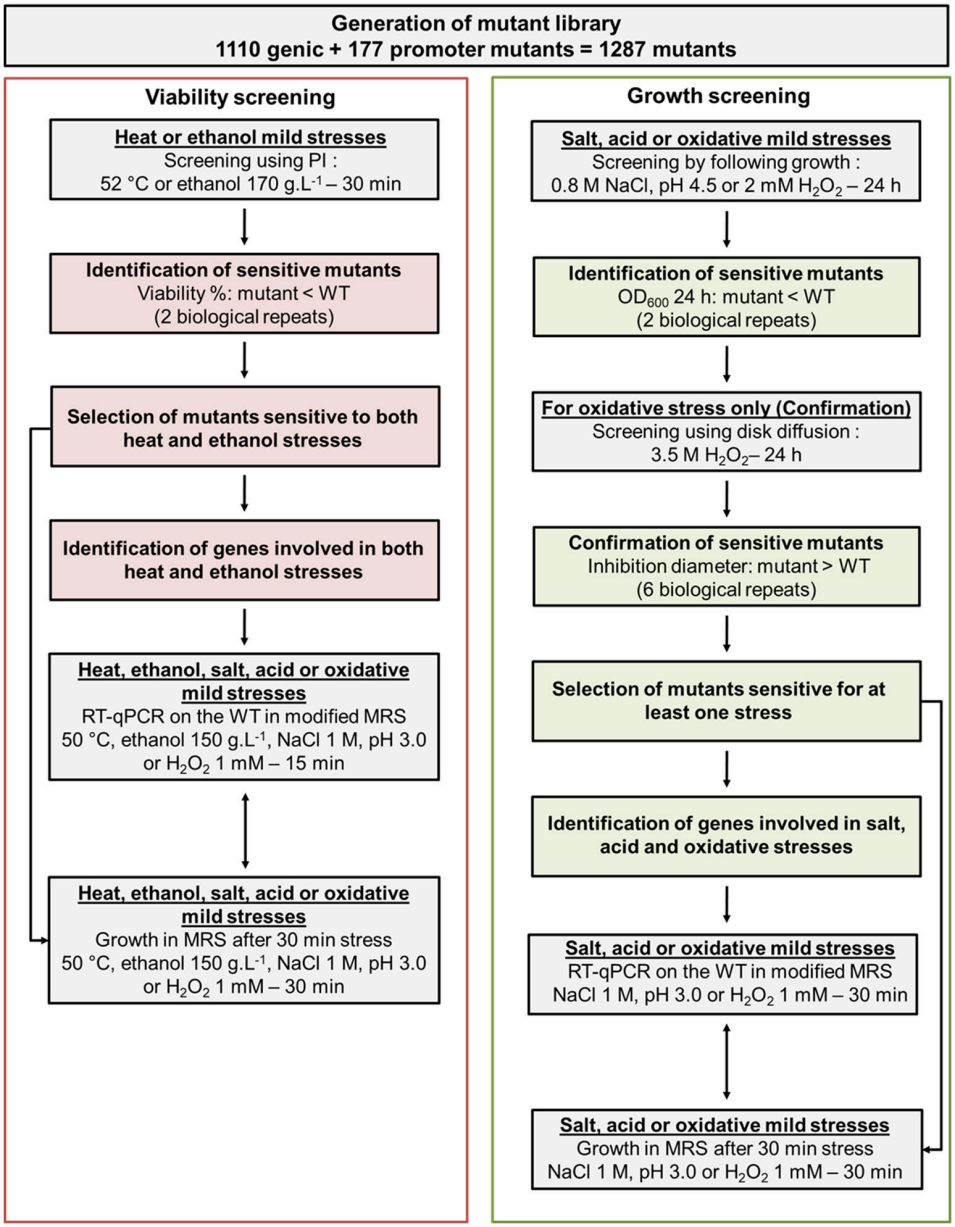
Organizational chart of the screening strategy to identify genetic factors involved in *L. paracasei* response to five monofactorial mild stresses: heat, ethanol, salt, acid, and oxidative.

In addition to the 1,110 mutants that have already been screened in a previous study (Licandro-Seraut et al., 2014), we have selected 177 new mutants in this work. These new mutants allowed us to increase the potential of information from the screening. They were selected because their transposon was inserted in the putative promoter of a gene for which no genic mutant was present in the initial genic library. Thus, a library containing a total number of 1,287 *L. paracasei* mutants was screened for the ability to resist to mild thermic, ethanol, acid, osmotic, and oxidative stresses in comparison with their parental strain (wild type, WT) as described in section Materials and Methods. The global screening strategy is shown in Figure 1.

Initially, we aimed to measure mutant viability using propidium iodide (PI) in 96-well plates for all types of stress since PI presents the advantage of a direct detection and a high sensitivity. For each stress, a range of conditions (intensity, time) was applied to WT resting cells (in phosphate buffer) to determine mild stress conditions, i.e., the highest intensity condition for which *L. paracasei* WT was at least 80% viable. A correlation between viabilities obtained by PI method and plate count agar method (CFU counts) was made for each stress with the WT. It demonstrated that PI staining was a relevant method for heat and ethanol stresses, but not for salt, acid, and oxidative stresses. Viabilities were 91 ± 7% (CFU counts) vs. 70 ± 1% (PI counts) for a 2 M NaCl stress, 94 ± 5% (CFU counts) and 76 ± 1% (PI counts) for a pH 2.4 stress, 41 ± 9% (UFC counts) and 84 ± 0% (PI counts) for a 3.5 mM H_2_O_2_ stress.

The following parameters were retained for the screening: 52°C during 30 min for heat stress and 170 g.L^−1^ ethanol during 30 min for ethanol stress. The WT viability was 82% (±1%) for heat stress and 84% (±2%) for ethanol stress (six biological repeats).

As an alternative, the three other stresses were applied at the beginning of the WT growth and their sensitivity was determined according to the growth inhibition triggered by the stress. Mild stress conditions were defined as the highest intensity condition for which the WT OD_600 nm_ (24 h) was less or equal to the 80% of OD_600 nm_ (24 h) in absence of stress. The retained parameters were pH 4.5, 0.8 M NaCl, and 2 mM H_2_O_2_ for acid, salt and oxidative stresses respectively. When WT strain was exposed to these stresses, OD_600 nm_ (24 h) was 1.33 (±0.03) for acid stress, 1.00 (±0.05) for salt stress, 1.31 (±0.03) for oxidative stress whereas it reached 1.73 (±0.05) in the control condition (MRS). For oxidative stress, as standard derivations were high for some mutants, the 23 selected mutants were subjected to diffusion test with 3.5 M H_2_O_2_ in order to confirm and quantify the sensitivity of each mutant for oxidative stress. Mutants were considered sensitive if their inhibition diameters were significantly higher than that of the WT (*p* < 0.05, six biological repeats).

### Identification of genes involved in responses to heat and ethanol mild stresses by viability screening

**Figure 2:**
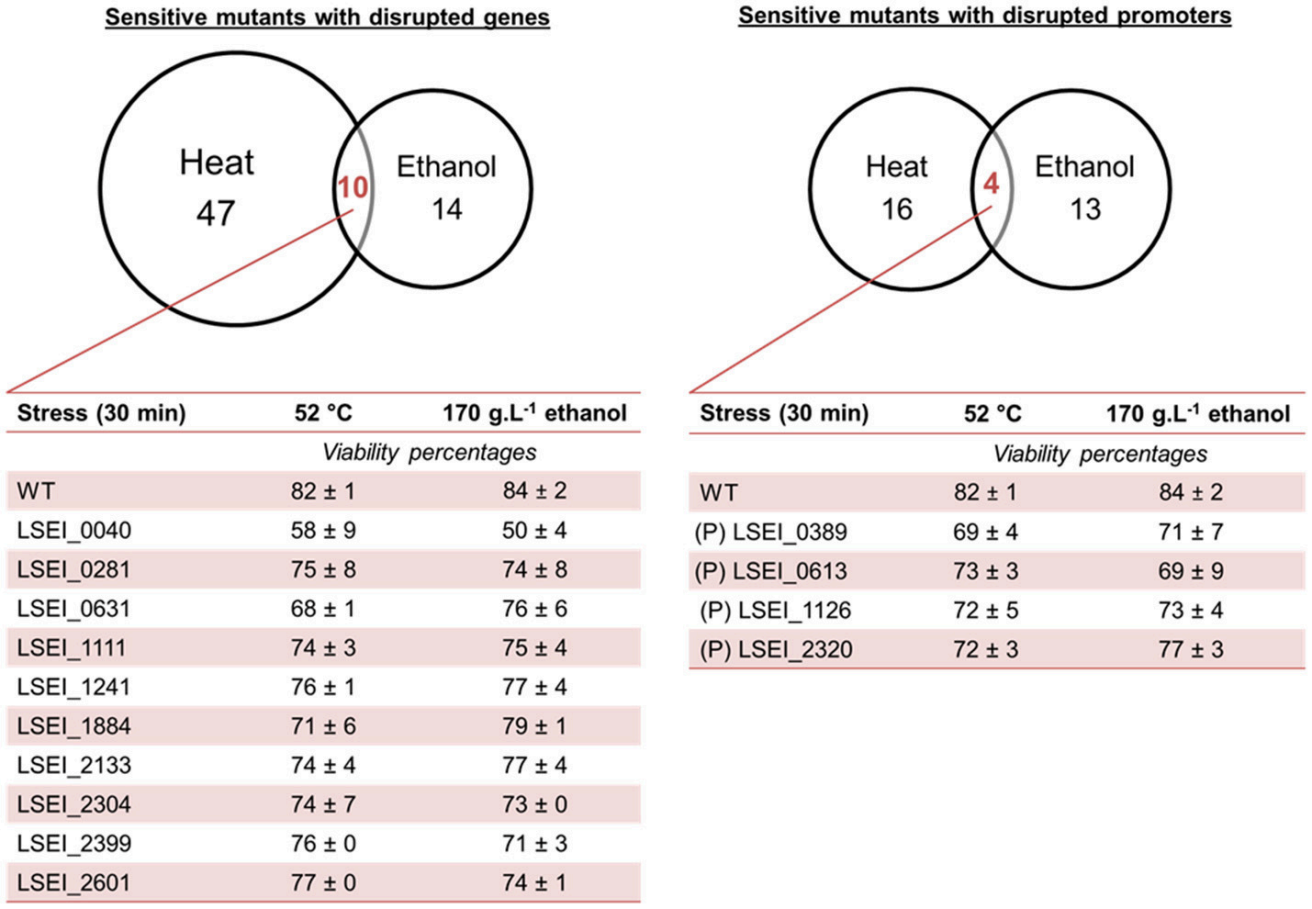
Venn diagram of *L. paracasei* mutants sensitive to heat (52°C, 30 min) and ethanol (170 g.L^−1^, 30 min) mild stresses. Corresponding viabilities using propidium iodide labeling are indicated only for mutants sensitive to the 2 stresses (at least 2 biological repeats). (P), intergenic insertion mutant (putative promoter). The predictive function of each gene is reported in Table 5.

The mutant library was subjected independently to heat stress and ethanol stress as described before. Viability analysis revealed 104 sensitive mutants including 71 genic and 33 intergenic transposon mutants (Figure 2). Mean viabilities ranged from 49 to 80% for sensitive mutants (Table S2). A large majority of mutants, 61 genic and 29 intergenic, were sensitive to only one stress condition (heat or ethanol). These disrupted genes encode for various predicted functions such as transcriptional regulation, amino acid transport and metabolism, carbohydrate transport and metabolism, membrane biogenesis, and hypothetical proteins. The most sensitive mutants were LSEI_0656 (P) (putative DNA-entry nuclease), LSEI_0824 (P) (putative hypothetical protein), and LSEI_2289 (putative hydrolase) for heat stress, LSEI_0221 (putative D-alanyl-D-alanine carboxypeptidase) and LSEI _2733 (putative L-xylulose-5-phosphate 3-epimerase) for ethanol stress, with viability lower than 70%. The 14 mutants sensitive to the two stresses are disrupted in cell wall/membrane genes such as putative ABC transporters, PTS system, membrane enzymatic function, but also in putative transposase genes, in a putative nucleotide and carbohydrate metabolism gene, and in putative genes of unknown function (Figure 2). LSEI_0040 (putative hypothetical protein) was the most sensitive mutant in this category. These genes could be attributed to general stress response.

### Identification of genes involved in response to salt, acid, and oxidative mild stresses by growth inhibition screening

**Figure 3:**
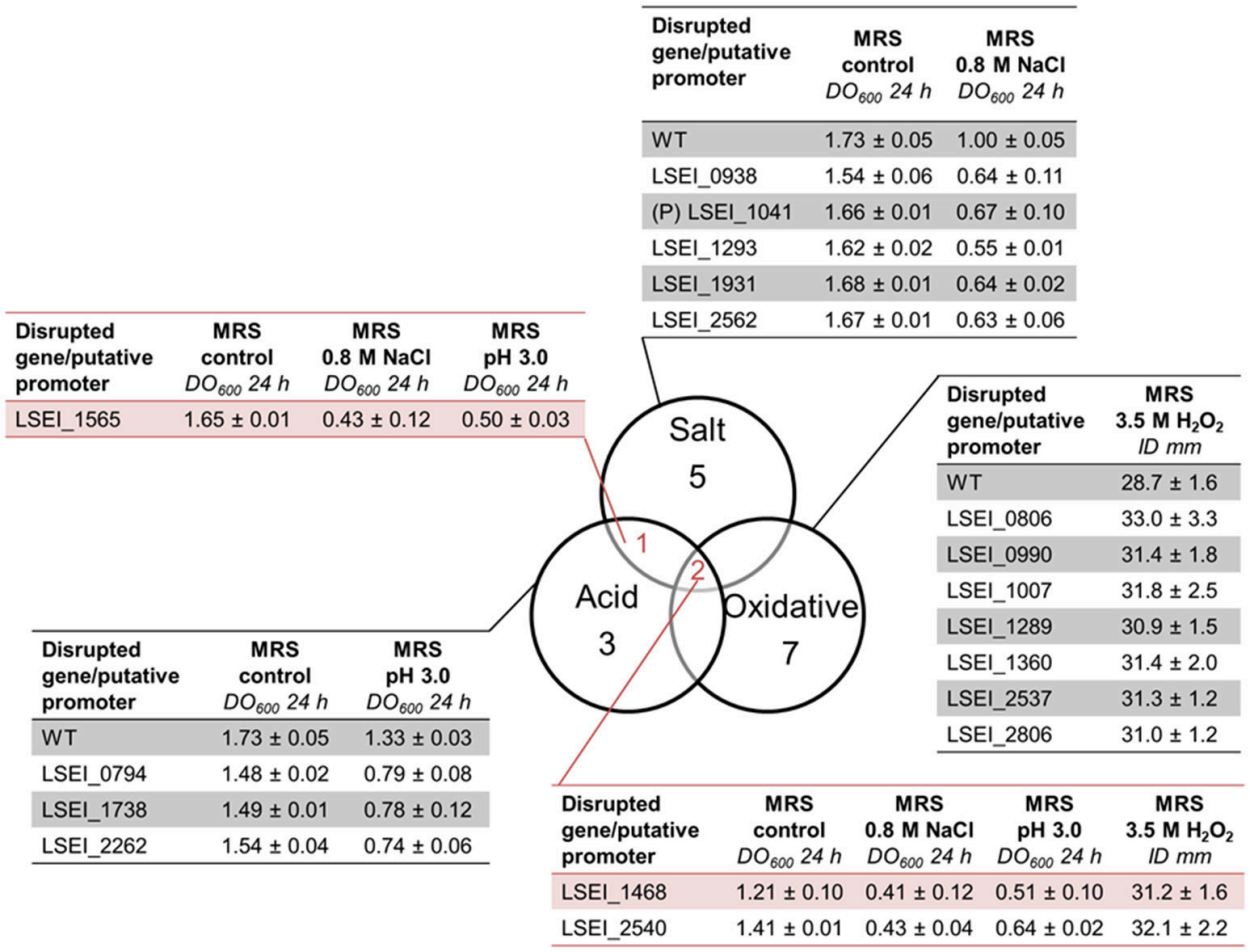
Venn diagram of *L. paracasei* mutants sensitive to salt (0.8 M NaCl, 24 h), acid (pH 4.5, 24 h), and/or oxidative (3.5 M H_2_O_2_, 24 h) mild stresses, their corresponding OD_600_ 24 h and their inhibition diameters (ID). (P), putative promoter. The predictive function of each gene is reported in Table 5.

The mutant library was subjected independently to salt stress (0.8 M NaCl), acid stress (pH 4.5), and oxidative stress (2 mM H_2_O_2_) during a 24 h-growth as described before. The screening resulted in detection of eight mutants for salt stress, six mutants for acid stress, and 23 mutants for oxidative stress (Table S3). For the sensitive mutants, DO_600_ (24 h) were 1.5–7 times lower than that of the WT. In a second round, oxidative mutants were screened on MRS plates with an H_2_O_2_ gradient. Among the 23 mutants initially selected, nine mutants displayed a significant sensitivity (*p* < 0.05) and were selected as sensitive (Figure 3). The mean final inhibition diameter ranged from 31 to 33 mm compared to 29 mm for the WT. Most of mutants were sensitive to only one of the three stresses. Their disrupted genes encode for various predicted functions such as Zn protease, D-alanine activating enzyme, ABC transporter for acid stress, ABC transporter, DNA binding response regulator, phosphoglycerate mutase for salt stress and ABC transporter, cysteine desulfurase, nucleotidase for oxidative stress. The most sensitive mutants were LSEI_1468 (putative ribonucleotide reductase), LSEI_1565 (DnaK), LSEI_2540 (putative Zn protease) with an OD_600_ (24 h) lower than 50% of that of the WT. Only three genes seemed to be involved in response to several stresses: LSEI_1468 and LSEI_2540 for the three stresses and LSEI_1565 for salt and acid stresses. So genetic responses to salt, acid, and oxidative mild stresses seem rather specific of the type of stress applied.

### Transcriptomic analysis of genes involved in acid, salt, or oxidative stresses

In order to expand the analysis, transcriptomic analysis of *L. paracasei* WT was performed on the 18 genes identified after the growth inhibition screening. WT was subjected to 15-min mild stresses (1 M NaCl for salt stress, pH 3.0 for acid stress, and 1 mM H_2_O_2_ for oxidative stress) and a culture without stress was used as the reference (Table 1). In parallel, for the 18 corresponding mutants, growth inhibition was measured after 30-min salt, acid and oxidative stresses of same intensity than for transcriptomics (Table 2). The growth of nine mutants was more inhibited than the WT after a stress application, whereas the growth of the 18 mutants was comparable to the WT in absence of stress. Among the 18 genes, six were upregulated for the stress for which the corresponding mutant was sensitive: LSEI_1468 (putative ribonucleotide reductase) and LSEI_2262 (putative hypothetical protein) for salt and oxidative stresses, LSEI_0794 (putative D-alanine-activating enzyme) and LSEI_1565 (DnaK) for salt stress, LSEI_2540 (putative Zn protease) for oxidative stress, LSEI_0938 (putative phosphate ABC transporter) for acid stress. The most up-regulated genes -with values over 3.0- were LSEI_0938 for acid stress (3.35 ± 0.69), LSEI_0794 and LSEI_2262 for salt stress (3.43 ± 0.93 and 3.99 ± 1.12) and LSEI_1565 for oxidative stress (3.21 ± 1.01).

**Table 1:** Differentially expressed genes in *L. paracasei* after acid, salt or oxidative stress among the 18 genes for which a corresponding mutant has been identified as sensitive for at least one of these stress conditions.

**Gene**	**Predicted function**	**Acid stress**	**Salt stress**	**Oxidative stress**
		**pH 3.0 15 min**	**1 M NaCl 15 min**	**1 mM H_2_O_2_ 15 min**
LSEI_0794	D-alanine-activating enzyme	1.51 ± 0.28	3.43 ± 0.93^*^	−1.21 ± 0.35
LSEI_0806	HP	1.81 ± 0.40	2.60 ± 0.50^*^	1.58 ± 0.44
LSEI_0938	Phosphate ABC transporter	3.35 ± 0.69^*^	1.01 ± 0.25	1.34 ± 0.39
LSEI_0990	Sugar ABC transporter	1.05 ± 0.25	−2.64 ± 0.63^*^	1.44 ± 0.48
LSEI_1007	Spermidine/putrescine ABC transporter	−2.33 ± 0.54^*^	−4.05 ± 1.13^*^	1.35 ± 0.52
LSEI_1041	DNA binding response regulator	1.35 ± 0.29	1.41 ± 0.31	1.22 ± 0.38
LSEI_1289	Cysteine desulfurase	−1.18 ± 0.25	−1.23 ± 0.27	−1.10 ± 0.34
LSEI_1293	Phosphoglycerate mutase	−1.17 ± 0.25	−1.34 ± 0.33	1.22 ± 0.36
LSEI_1360	5'-nucleotidase	**1.96** ±, 0.50^*^	−1.01 ± 0.30	1.57 ± 0.51
LSEI_1468	Ribonucleotide reductase	1.37 ± 0.33	2.45 ± 0.56^*^	2.08 ± 0.57^*^
LSEI_1565	DnaK	−1.29 ± 0.18	2.04 ± 0.77^*^	3.21 ± 1.01^*^
LSEI_1738	Peptide ABC transporter	−1.70 ± 0.37^*^	−1.62 ± 0.52	1.88 ± 0.62
LSEI_1931	HP	1.26 ± 0.42	1.18 ± 0.52	1.11 ± 0.36
LSEI_2262	HP	1.51 ± 0.41	3.99 ± 1.12^*^	1.99 ± 0.68^*^
LSEI_2537	Cell surface protein	−1.13 ± 0.18	2.78 ± 0.72^*^	2.03 ± 0.51
LSEI_2540	ATP-dependent Zn protease	1.00 ± 0.25	1.51 ± 0.45	1.96 ± 0.64^*^
LSEI_2562	DNA/RNA helicase	−2.86 ± 0.82^*^	−1.41 ± 0.34	−2.23 ± 0.74
LSEI_2806	HP	1.47 ± 0.29	2.69 ± 0.88	1.07 ± 0.39

All stresses were performed at 37°C during 15 min. Relative gene expressions were calculated using 2^−ΔΔCt^ method. For MRS control condition, a gene expression value of 1.0 was attributed and genes expressions in stress condition were calculated in function of this value. Positive values (>1.0) represent upregulation and negative values (<1.0) represent downregulation.

* *Significant changes in gene expression (p < 0.05) (in bold) compared to the not stressed culture (4 biological replicates). HP, Hypothetical protein*.

**Table 2:** Growth percentages after acid, salt, or oxidative stress, for the 18 mutants identified as sensitive for at least one of these stress conditions.

**Mutant**	**Mean percentage of growth for stress condition relative to control condition**			
	**MRS (control) (%)**	**Acid stress pH 3.0 (%)**	**Salt stress 1 M NaCl (%)**	**Oxidative stress 1 mM H_2_O_2_ (%)**
WT	100	91	75	78
Selection of sensitive mutants		<82	<69	<72
LSEI_0794	100	93	**68**	82
LSEI_0806	100	95	75	80
LSEI_0938	100	**81**	75	76
LSEI_0990	100	87	77	75
LSEI_1289	100	95	78	76
LSEI_1007	100	95	81	82
(P) LSEI_1041	100	83	**63**	**68**
LSEI_1293	100	88	84	90
LSEI_1360	100	93	84	88
LSEI_1468	100	**78**	**68**	**70**
LSEI_1565	100	88	**68**	76
LSEI_1738	100	87	70	**71**
LSEI_1931	100	**79**	82	79
LSEI_2262	100	**81**	**68**	**67**
LSEI_2537	100	93	79	80
LSEI_2540	100	**64**	**52**	**66**
LSEI_2562	100	88	76	81
LSEI_2806	100	95	83	81

*All stresses were performed at 37°C during 30 min in MRS. For MRS control condition, a 100% growth percentage was attributed for the mean OD600 7 h of the WT and the different mutants. Growth percentages in stress condition were calculated in function of the mean OD600 7 h in MRS and in stress condition. For each stress, growth percentages for selection of sensitive mutants were calculated using the mean OD600 7 h of the WT minus the value of two standard derivations. The values in bold correspond to sensitive mutants with a growth percentage in stress condition inferior to the growth percentage of the WT strain (two biological repeats). (P), putative promoter. The predictive function of each gene is reported in Table 5*.

### Transcriptomic analysis of genes involved in both heat and ethanol stresses

To study general stress response, the identification of the sensitive mutants for both heat and ethanol stresses (14 mutants) was complemented by transcriptomic analysis on the corresponding genes for all stresses. Transcriptomic analysis was performed on the WT subjected to 15-min stresses (150 g.L^−1^ for ethanol stress, 50°C for heat stress, 1 M NaCl for salt stress, pH 3.0 for acid stress, and 1 mM H_2_O_2_ for oxidative stress; Table 3). Among the 14 genes, eight were upregulated for the stress for which the corresponding mutant was sensitive (Table 4): LSEI_0281 (putative cell wall-associated hydrolase) and LSEI_1241 (putative transposase) for heat stress, LSEI_1884 (putative peptide ABC transporter) and LSEI_2320 (putative mucin binding protein) for salt stress, LSEI_1111 and LSEI_2304 (putative hypothetical proteins) for heat and salt stress, LSEI_0389 (putative pseudo gene) for heat and acid stress and LSEI_0631 for all stress conditions. For this last gene, encoding a putative PTS system, expressions were over 4.0 for heat (5.47 ± 3.07), salt (4.45 ± 1.11), acid (4.68 ± 0.77), and oxidative (10.20 ± 3.01) stresses. LSEI_1884 gene, encoding a putative peptide ABC transporter, was strongly upregulated only for salt stress (11.11 ± 3.43). Four genes, LSEI_0040 (putative hypothetical protein), LSEI_0389 (putative pseudo gene), LSEI_0613 and LSEI_1241 (putative transposases) were more upregulated for heat stress (value >3.0). LSEI_2399 (putative metal-dependent membrane protease) was upregulated especially for heat (5.43 ± 1.96) and acid (4.51 ± 1.07) stresses.

**Table 3:** Differentially expressed genes in *L. paracasei* after heat, ethanol, acid, salt, or oxidative stress, for the 14 genes for which a corresponding mutants has been identified as sensitive to both heat and ethanol stress conditions.

**Gene**	**Predicted unction**	**Heat stress**	**Ethanol stress**	**Salt stress**	**Acid stress**	**Oxidative stress**
		**50°C 15 min**	**150 g.L^−1^ 15 min**	**1M NaCl 15 min**	**pH 3.0 15 min**	**1 mM H_2_O_2_ 15 min**
LSEI_0040	HP	3.45 ± 1.55^*^	−5.90 ± 1.04^*^	−1.68 ± 0.48	1.19 ± 0.28	−1.06 ± 0.34
LSEI_0281	Cell wall-associated hydrolase	2.73 ± 0.60^*^	**–**1.30 ± 0.24	1.22 ± 0.39	1.19 ± 0.22	1.80 ± 0.51^*^
LSEI_0389	Sugar ABC transporter	3.77 ± 1.14^*^	−3.69 ± 0.68^*^	1.56 ± 0.39	2.92 ± 0.50^*^	1.55 ± 0.46
LSEI_0613	Transposase	3.73 ± 1.61^*^	−4.62 ± 0.85^*^	1.08 ± 0.28	1.20 ± 0.19	1.32 ± 0.,41
LSEI_0631	Beta-glucoside- PTS system	5.47 ± 3.07^*^	2.13 ± 0.30^*^	4.45 ± 1.11^*^	4.68 ± 0.77^*^	10.2 ± 3.01^*^
LSEI_1111	HP	2.97 ± 1.74^*^	−1.21 ± 0.22	1.79 ± 0.56^*^	2.02 ± 0.48^*^	1.22 ± 0.36
LSEI_1126	glucose-6-phosphate isomerase	2.21 ± 0.61^*^	−5.48 ± 0.91^*^	−1.08 ± 0.46	1.16 ± 0.19	1.97 ± 0.56^*^
LSEI_1241	Transposase	3.25 ± 1.74^*^	−1.04 ± 0.16	1.15 ± 0.37	2.27 ± 0.57^*^	1.24 ± 0.35
LSEI_1884	Peptide ABC transporter	6.59 ± 3.65	−1.38 ± 0.38	11.11 ± 3.43^*^	1.39 ± 0.37	1.17 ± 0.35
LSEI_2133	ADP-ribose pyrophosphatase	2.47 ± 1.07	−1.62 ± 0.34^*^	−1.37 ± 0.38	1.82 ± 0.59^*^	−1.15 ± 0.29
LSEI_2304	HP	2.12 ± 0.77^*^	−3.97 ± 0.79^*^	1.67 ± 0.43^*^	−1.52 ± 0.37	1.01 ± 0.30
LSEI_2320	Mucin binding protein	1.66 ± 0.48	−**10.40** ±2.08^*^	2.35 ± 0.58^*^	1.22 ± 0.22	1.18 ± 0.41
LSEI_2399	Metal-dependent membrane protease	5.43 ± 1.96^*^	−2.90 ± 0.51^*^	1.73 ± 0.50^*^	4.51 ± 1.07^*^	1.58 ± 0.36^*^
LSEI_2601	Multidrug ABC transporter	1.65 ± 0.68	−2.70 ± 0.51^*^	−1.31 ± 0.26	−1.04 ± 0.20	1.82 ± 0.78

All stresses were performed at 37°C during 15 min. Relative gene expressions were calculated using 2^−ΔΔCt^ method. For MRS or phosphate buffer control condition, a gene expression value of 1.0 was attributed and genes expressions in stress condition were calculated in function of this value. Positive values (>1.0) represent upregulation and negative values (<1.0) represent downregulation.

* *Significant changes in gene expression (p < 0.05) (in bold) compared to the not stressed culture (four biological replicates). HP, Hypothetical protein*.

**Table 4:** Growth percentages after heat, ethanol, acid, salt, or oxidative mild stress, for the 14 mutants identified as sensitive to both heat and ethanol stress conditions.

	**Mean percentage of growth for stress condition relative to control condition**					
**Mutant**	**MRS (control) (%)**	**Heat stress 50°C (%)**	**Ethanol stress 150 g.L^−1^ (%)**	**Salt stress 1M NaCl (%)**	**Acid stress pH 3.0 (%)**	**Oxidative stress 1 mM H_2_O_2_ (%)**
WT	100	66	75	75	91	78
Selection of sensitive mutants		<60	<69	<69	<82	<72
LSEI_0040	100	68	73	**56**	97	80
LSEI_0281	100	62	72	72	97	78
(P) LSEI_0389	100	**57**	**68**	**54**	**77**	**68**
(P) LSEI_0613	100	65	71	**63**	85	73
LSEI_0631	100	**52**	**63**	**66**	**78**	**71**
LSEI_1111	100	**51**	**63**	**66**	85	**68**
(P) LSEI_1126	100	78	**68**	78	83	77
LSEI_1241	100	**58**	69	**53**	94	74
LSEI_1884	100	**55**	69	**65**	85	74
LSEI_2133	100	**59**	70	74	92	75
LSEI_2304	100	**58**	73	70	95	75
(P) LSEI_2320	100	69	**66**	**61**	**80**	72
LSEI_2399	100	67	74	74	93	82
LSEI_2601	100	60	73	73	89	73

*All stresses were performed at 37°C during 30 min in MRS. For MRS control condition, a 100% growth percentage was attributed for the mean OD600 7 h of the WT and the different mutants. Growth percentages in stress condition were calculated in function of the mean OD600 7 h in MRS and in stress condition. For each stress, growth percentages for selection of sensitive mutants were calculated using the mean OD600 7 h of the WT minus the value of two standard derivations. The values in bold correspond to sensitive mutants with a growth percentage in stress condition inferior to the growth percentage of the WT strain (two biological repeats). (P), putative promoter. The predictive function of each gene is reported in Table 5*.

## Discussion

### Development of the mutant screening

In this study, a transposon mutant library of 1,287 mutants was exposed to five monofactorial mild stresses in order to identify genetic determinants involved in bacterial adaptation. The first part of the work was to develop a suitable screening method which should be easy and fast but also and mostly accurate. We observed that viability determinations using propidium iodide as the marker of mortality, although very easy and fast to carry out, were aberrant for studying salt, acid, and oxidative stresses. Thus we developed growth inhibition screening for these stresses. Previous studies have also reported that CFU counts and fluorescence staining methods were sometimes not correlated in particular when adherent bacteria were harvested using sonication, which could lead to a higher number of colonies on plates (Hannig et al., 2007; Tawakoli et al., 2013). For oxidative stress, as observed by Zotta et al. (2012), bacteria were not stained with PI. Maybe this kind of stress does not immediately affect membrane integrity.

### Comparison of mutagenesis and transcriptomic approaches for stress response

To complete the results obtained after mutant screenings, transcriptomic analyzes were performed on selected genes. Among the 32 genes required in stress response and selected for transcriptomics, 10 genes were not differentially expressed in the stressed WT. This result strengthens the scope of global reverse genetics compared to global transcriptomics since many genes may be essential for a function but constitutively expressed. Other studies have demonstrated that some genes can be required for stress resistance without exhibiting transcriptomic changes. It is particularly the case for genes encoding monocistronic transcriptional regulators (Tran et al., 2008). Recently, Price et al. (2013) have shown that there was only little correlation between gene upregulation and mutant sensitivity in their bacterial models.

### Genes involved in general stress response

Mutant library screening led to the identification of 20 genes and 5 putative promoters involved in multiple stress response (at least two stresses; Table 5). For 15 of them, we did not find any literature reporting their role in stress response (Tables S2, S3) thus they can be considered as new multiple stress response determinants. Most are specific of the *L. casei/paracasei/rhamnosus* group. On the contrary, the putative ABC transporter LSEI_1884 and the putative response regulator of a two component system LSEI_1041 caught our attention because they are well conserved among Gram-positive bacteria. On the other hand, four genes, LSEI_0631 (putative beta-glucoside-specific PTS system), LSEI_1111 (putative hypothetical protein), LSEI_0389 (putative pseudo gene), and LSEI_2320 (putative mucin binding protein) can be considered as important general stress determinants because involved in response to 4 or 5 stress conditions (Table 5). The putative PTS system beta-glucoside-specific transporter (LSEI_0631) was the only one upregulated for all stress conditions, which perfectly matches the screening results. The PTS mannose system is one of the main sugar transporters for LAB. Several authors have reported a link between PTS systems and stress response for *Lactobacillus* genus. *L. plantarum* mutant for *rpoN* displayed an impaired expression of the mannose PTS operon, which increased their sensitivity to peroxide (Stevens et al., 2010). The authors have assumed that the suppression of this transporter led to the diminution of glucose capture and energy that may explain the susceptibility to oxidative stress. A *L. casei* resistant mutant for acid stress obtained by adaptation had a higher PTS activity than the WT after 1 h at pH 5.0 (Wu et al., 2012). The authors suggest that this results may lead to higher level of ATP via glycolysis which allows maintaining intracellular pH. LSEI_1111 had no function assigned. Transcriptomic analysis of LSEI_0389, encoding a putative pseudo-gene, demonstrated that it was transcribed and upregulated for heat and acid stresses. LSEI_2320 possesses three mucin binding domains (MucBP) which are involved in cell adhesion (Munoz-Provencio et al., 2012; Yamasaki-Yashiki et al., 2017). It has never been reported as determinant of stress response before.

**Table 5:** *L. paracasei* genes and putative promoters involved in mild stress response.

**Gene/Promoter**	**Predicted function**	**Stress**				
(P) LSEI_0020	Surface antigen	H				
LSEI_0040	HP	H	E	S		
LSEI_0082	HP	H				
LSEI_0100	Diaminopimelate epimerase		E			
LSEI_0118	HP	H				
(P) LSEI_0120	N-acetylmuramic acid 6-phosphate etherase (murQ)	H				
(P) LSEI_0175	Oligopeptide ABC transporter		E			
LSEI_0221	D-alanyl-D-alanine carboxypeptidase		E			
LSEI_0242	Mn/Zn ABC transporter	H				
LSEI_0281	Cell wall-associated hydrolase	H	E			
LSEI_0289	Sugar ABC transporter	H				
LSEI_0324	Transcriptional antiterminator	H				
LSEI_0330	Transcriptional regulator	H				
(P) LSEI_0389	Pseudo gene	H	E	S	A	O
LSEI_0394	Transcriptional regulator	H				
LSEI_0428	Transcriptional regulator	H				
(P) LSEI_0439	Glutamine synthetase		E			
(P) LSEI_0452	Pseudo gene	H				
LSEI_0460	DNA-binding response regulator (TCS)	H				
LSEI_0492	HP	H				
(P) LSEI_0550	Holin-like toxin	H				
(P) LSEI_0613	Transposase, IS30 family	H	E	S		
LSEI_0631	Beta-glucoside-specific PTS system	H	E	S	A	O
(P) LSEI_0652	NADPH:quinone reductase related Zn-dependent oxidoreductase		E			
(P) LSEI_0656	DNA-entry nuclease	H				
LSEI_0661	Glycerol-3-phosphate dehydrogenase	H				
(P) LSEI_0723	Membrane protein		E			
LSEI_0756	Hydrolase of the alpha/beta superfamily protein	H				
LSEI_0794	D-alanine-activating enzyme			S	A	
LSEI_0806	HP					O
(P) LSEI_0824	HP	H				
(P) LSEI_0916	DNA segregation ATPase protein	H				
LSEI_0938	Phosphate ABC transporter			S	A	
LSEI_0990	Sugar ABC transporter					O
LSEI_1007	Spermidine/putrescine ABC transporter	H				O
(P) LSEI_1041	DNA binding response regulator (TCS)	H		S		O
(P) LSEI_1091	HP		E			
LSEI_1111	HP	H	E	S		O
LSEI_1115 (P)	Acetyltransferase		E			
(P) LSEI_1126	Glucose-6-phosphate isomerase	H	E			
LSEI_1128	HP	H				
(P) LSEI_1133	Transposase, IS30 family		E			
LSEI_1178	Methionine import ATP-binding protein (MetN)		E			
(P) LSEI_1236	HP	H				
LSEI_1241	Transposase	H	E	S		
LSEI_1289	Cysteine desulfurase					O
LSEI_1293	Phosphoglycerate mutase			S		
(P) LSEI_1332	Elongation factor Tu	H				
LSEI_1360	5′-nucleotidase					O
LSEI_1403	Tyrosine recombinase		E			
LSEI_1419	Signal transduction histidine kinase		E			
LSEI_1421	Permease	H				
(P) LSEI_1437	NUDIX family hydrolase	H				
LSEI_1450	Orotidine-5′-phosphate decarboxylase	H				
LSEI_1466	HP	H				
LSEI_1468	Ribonucleotide reductase			S	A	O
LSEI_1470	HP		E			
LSEI_1497	Metal-sulfur cluster biosynthetic enzyme	H				
LSEI_1543	HP	H				
LSEI_1565	DnaK			S	A	
LSEI_1566	GrpE		E			
LSEI_1580	Zinc metalloprotease	H				
(P) LSEI_1679	DNA-binding response regulator (TCS)		E			
LSEI_1738	Peptide ABC transporter				A	O
(P) LSEI_1763	HP	H				
LSEI_1821	HP	H				
LSEI_1884	Peptide ABC transporter	H	E	S		
LSEI_1931	HP			S	A	
LSEI_1945	Phage protein	H				
LSEI_1951	Phage protein		E			
LSEI_1970	HP		E			
LSEI_2024	HP	H				
LSEI_2033	Transcriptional regulator	H				
LSEI_2049	Capsular polysaccharide biosynthesis protein	H				
(P) LSEI_2059	Transcriptional regulator		E			
LSEI_2082	Exonuclease	H				
LSEI_2091	Integrase	H				
LSEI_2096	HP	H				
LSEI_2129	Esterase		E			
LSEI_2133	ADP-ribose pyrophosphatase	H	E			
LSEI_2162	Asparagine synthase	H				
LSEI_2196	HP	H				
LSEI_2215	Protein tyrosine/serine phosphatase	H				
LSEI_2262	HP	H		S	A	O
LSEI_2269	Trancriptional regulator	H				
LSEI_2289	Hydrolase of the alpha/beta surperfamily protein	H				
LSEI_2304	HP	H	E			
LSEI_2317	Membrane protein	H				
(P) LSEI_2320	Mucin binding protein	H	E	S	A	
LSEI_2399	Metal-dependent membrane protease	H	E			
LSEI_2439	HP	H				
LSEI_2533	tRNA-dihydrouridine synthase	H				
LSEI_2537	Cell surface protein		E			O
LSEI_2540	ATP-dependent Zn protease			S	A	O
(P) LSEI_2548	Peptidyl-tRNA hydrolase	H				
LSEI_2562	DNA/RNA helicase			S		
LSEI_2565	HP	H				
(P) LSEI_2579	Large-conductance mechanosensitive channel (mscL)		E			
(P) LSEI_2583	HP		E			
LSEI_2601	Multidrug ABC transporter	H	E			
(P) LSEI_2606	ADP-ribose pyrophosphatase		E			
LSEI_2613	HP	H				
LSEI_2616	Polyphosphate kinase	H				
LSEI_2619	SAM-dependent methyltransferase	H				
LSEI_2626	Peptide ABC transporter	H				
LSEI_2697	Pseudo gene	H				
(P) LSEI_2698	Transposase, IS30 family		E			
(P) LSEI_2716	HP	H				
LSEI_2733	L-xylulose-5-phosphate 3-epimerase		E			
LSEI_2734	Transcriptional regulator		E			
LSEI_2739	Zn-dependent hydrolase	H				
LSEI_2787	NADPH:quinone reductase related Zn-dependent oxidoreductase	H				
LSEI_2806	HP					O
LSEI_2880	Membrane protein	H				
(P) LSEI_2884	Esterase/lipase	H				
LSEI_A15	HP	H				
LSEI_r1832	23S ribosomal RNA		E			
(P) LSEI_t0720	tRNA	H				
Total	77	41	18	11	15	

*Genes and putative promoters in red were shown to be involved in general stress response. H, Heat stress, E, Ethanol stress, S, Salt stress, A, Acid stress, O, Oxidative stress. (P), putative promoter, HP, Hypothetical protein*.

Moreover, we have observed that 11 of the 25 identified genes were also involved in multiple stress response at the transcriptomic level (genes in red in Table 6). This is the case of the chaperone *dnaK* (LSEI_1565) which is known to be involved in various LAB stress responses (van de Guchte et al., 2002). Also, the expression of genes involved in membrane modification such as LSEI_0281 (cell wall-associated hydrolase) and LSEI_2399 (predicted metal-dependent membrane protease) was increased and highlighted that cell envelope feature was essential for multiple stress response. The phosphoglucose isomerase gene (LSEI_1126, putative glucose-6-phosphate isomerase) was upregulated for heat, ethanol, and oxidative stresses. This gene was upregulated during exposure of *L. plantarum* to manganese (Tong et al., 2017).

**Table 6:** Summary of genes involved in general and specific stress response in function of the genetic approach used (mutagenesis or transcriptomics).

**Gene**		**Mutagenesis**					**Transcriptomic**				
**AMINO ACID TRANSPORT/METABOLISM**											
LSEI_1007		H				O					
LSEI_1289						O					
**CARBOHYDRATE TRANSPORT/METABOLISM**											
LSEI_0631		H	E	S	A	O	H	E	S	A	O
LSEI_0990						O					
(P) LSEI_1126		H	E				H	E			O
LSEI_1293				S							
**NUCLEOTIDE METABOLISM**											
LSEI_1360						O				A	
LSEI_1468				S	A	O			S		O
LSEI_2133		H	E							A	
**MOLECULAR CHAPERONE**											
LSEI_1565				S	A				S		O
LSEI_2540				S	A	O					O
**RESPONSE REGULATOR**											
(P) LSEI_1041		H		S		O					
LSEI_0938				S	A					A	
LSEI_1738					A	O					
LSEI_1884		H	E	S					S		
LSEI_2601		H	E								
**CELL WALL/MEMBRANE BIOGENESIS**											
LSEI_0281		H	E				H				O
LSEI_0794				S	A				S		
LSEI_2399		H	E				H	E	S	A	O
LSEI_2537			E			O			S		
**DNA**											
LSEI_2562				S							
(P) LSEI_0613		H	E	S			H				
LSEI_1241		H	E	S			H			A	
**UNKNOWN FUNCTION**											
LSEI_0040		H	E	S			H				
(P) LSEI_0389		H	E	s	A	O	H			A	
LSEI_0806						O			S		
LSEI_1111		H	E	S		O	H		S	A	
LSEI_1931				S	A						
LSEI_2262		H		S	A	O			S		O
LSEI_2304		H	E				H		S		
(P) LSEI_2320		H	E	S	A				S		
LSEI_2806						O					

*Genes in red were shown to be involved in general stress response using both transposon mutagenesis and transcriptomic analysis. Genes in gray represent potential transcriptomic biomarkers for one stress condition whose disruption lead to a sensitive mutant for the same stress. H, Heat stress, E, Ethanol stress, S, Salt stress, A, Acid stress, O, Oxidative stress. (P), putative promoter. The predictive function of each gene is reported in Table 5*.

Our results have shown that the salt and oxidative stresses increased the expression of a putative ribonucleotide reductase gene (LSEI_1468, RNR). On the contrary, under bile stress, a decrease of RNR expression was observed in other *Lactobacillus* (Burns et al., 2010; Koskenniemi et al., 2011). The hypothesis would be that this decrease was probably a consequence of a reduced growth rate and a decrease of DNA replication. Mutagenesis and transcriptomics highlight the involvement of two putative transposase genes (LSEI_1241 and LSEI_0613) in several stresses. Similarly, 12 other transposase genes were upregulated during acid adaptation at pH 4.5 in *L. casei* (Broadbent et al., 2010). Authors have assumed that stimulation of transposition could provide an evolutionary advantage to the host.

### Genes involved in specific stress responses

Among the 1,287 screened mutants, 118 were sensitive to at least one stress condition. Comparison of stress response indicates that genes are generally specific to one stress condition (93 genes, Table 5). All of these specific genes are relevant as indicators of a particular stress (i.e., stress markers). A large part of them have never been reported as required for stress response. Hence, to our knowledge, we have identified 47 *L. casei/paracasei/rhamnosus* group genes, 22 *Lactobacillus* specific genes and 23 Gram-positive bacteria genes as new genes for specific stress responses (Tables S2, S3). Remarkably, we have reported the involvement of LSEI_1945 and LSEI_1951 which encode putative phage proteins, a functional family that has never been reported to act during stress response.

### Identifications of stress markers

Cellular biomarkers are useful tools to predict the impact of environmental changes on bacterial robustness and survival. Generally, applications of stress markers are rather based on upregulations at the transcriptomic and/or proteomic level. For instance, in *L. casei*, a work has investigated the potential markers of bile tolerance by comparison of the proteomic profiles of six strains with different bile tolerance levels (Hamon et al., 2012). We have analyzed transcription of some stress-specific genes on *L. paracasei* WT when subjected to monofactorial mild stresses. We have selected genes as stress markers because they were upregulated specifically for one stress condition and their disruption led to an increased sensitivity to the same stress. Three genes (LSEI_0794, LSEI_1884 and LSEI_2320) were identified for NaCl stress, two genes (LSEI_0040 and LSEI_0613) for heat stress, one gene (LSEI_0938) for acid stress, and one gene (LSEI_2540) for oxidative stress (genes in gray in the Table 6). Interestingly, all biomarkers identified except LSEI_0613 (a transposase, IS*30* family) are related to cell wall functions or localization (LSEI_0040 is a gene of unknown function with several transmembrane domains). LSEI_0794 (putative D-alanine-activating enzyme), _1884 (putative peptide ABC transporter), _0613 (putative transposase), _0938 (putative phosphate ABC transporter), and _2540 (putative ATP dependant Zn protease) present a higher potential as biomarker since they are not restricted to the *L. paracasei* group genomes.

Some functions have already been associated to stresses. Firstly, the *dlt* operon (the LSEI_0793–0797ortholog), responsible for D-alanine incorporation into teichoic acids, is upregulated during bile and heat stresses in *L. plantarum* (Xie et al., 2004; Bron et al., 2006). Secondly, in *L. plantarum, ftsH* mutation (LSEI_2540 ortholog, ATP dependent Zn protease) reduced the growth rate in physiological conditions, and the growth defect became more important under stress (Bove et al., 2012). Thirdly, several studies have reported the involvement of ABC transporters in LAB in response to bile or osmotic stresses (Bouvier et al., 2000; Hamon et al., 2011; Alcantara and Zuniga, 2012; Wang et al., 2015). Peptide transport systems do not only play a role in cell nutrition, but are also involved in various signaling processes (Detmers et al., 2001). In this study, LSEI_0938 and LSEI_1884 encode putative ABC transporters, the latter represents a strong marker of salt stress since the measured upregulation was the most important of this work (11.11 ± 3.43).

## Conclusion

This work explored the screening of a transposon mutant library of *L. paracasei* to identify genes required to face five monofactorial mild stresses. It was complemented by targeted transcriptomics on selected genes. Firstly, 118 genes whose mutation led to a sensitive mutant were identified. These genes were generally specific to one stress condition and most of them had never been reported to be involved in stress response or adaptation. They deserve to be studied in a more thorough way to explore LAB genetic stress response. At least half of them are conserved in *Lacobacillus* genus. Secondly, five of the seven genes identified as potential stress biomarkers are related to the cell wall and could be used as selective stress reporters of membrane injury. Finally, some of these genes whose expression was not regulated by stress would have escaped a transcriptomics or proteomics screening. The identified stress determinants and biomarkers contribute to the comprehension of stress responses and adaptation, including stresses encountered in microbial processes and in food matrices. They could be targets for a better control of growth performance and functional properties of starters in food. Hence, the selection of high performance strains could be achieved by screening strains for the presence of the target gene. In addition, the expression of the target gene could be monitored and would indicate the intensity of stress during food process. In the context of improving starters by stress adaptation, since most of the stress response genes identified here are stress-specific, we assume that the nature of the mild stress (or priming stress) has to be carefully selected in function of the perturbations during the process.

## Author contributions

AP, HS, HL, and J-FC: study conception and design. AP: experimentation and acquisition of data. AP and HL: analysis and interpretation of data. AP: drafting of manuscript. AP, HS, HL, and J-FC: critical revision. All authors read and approved the final manuscript.

### Conflict of interest statement

The authors declare that the research was conducted in the absence of any commercial or financial relationships that could be construed as a potential conflict of interest.

## References

[B1] Alcántara C. Zúñiga M. (2012). Proteomic and transcriptomic analysis of the response to bile stress of *Lactobacillus casei* BL23. Microbiology 158, 1206–1218. 10.1099/mic.0.055657-0 22322960

[B2] Andrade-Linares D. R. Lehmann A. Rillig M. C. (2016). Microbial stress priming: a meta-analysis: microbial priming to stress. Environ. Microbiol. 18, 1277–1288. 10.1111/1462-2920.13223 26768991

[B3] Beaufils S. Sauvageot N. Mazé A. Laplace J.-M. Auffray Y. Deutscher J.. (2007). The cold shock response of *Lactobacillus casei*: relation between HPr phosphorylation and resistance to freeze/thaw cycles. J. Mol. Microbiol. Biotechnol. 13, 65–75. 10.1159/000103598 17693714

[B4] Bouvier J. Bordes P. Romeo Y. Fourçans A. Bouvier I. Gutierrez C.. (2000). Characterization of OpuA, a glycine-betaine uptake system of *Lactococcus lactis*. J. Mol. Microbiol. Biotechnol. 2, 199–205. Available online at: https://www.caister.com/jmmb/tocs/v2n2toc.html 10939245

[B5] Bove P. Capozzi V. Garofalo C. Rieu A. Spano G. Fiocco D. (2012). Inactivation of the ftsH gene of *Lactobacillus plantarum* WCFS1: effects on growth, stress tolerance, cell surface properties and biofilm formation. Microbiol. Res. 167, 187–193. 10.1016/j.micres.2011.07.001 21795030

[B6] Broadbent J. R. Larsen R. L. Deibel V. Steele J. L. (2010). Physiological and transcriptional response of *Lactobacillus casei* ATCC 334 to acid Stress. J. Bacteriol. 192, 2445–2458. 10.1128/JB.01618-09 20207759 PMC2863488

[B7] Bron P. A. Molenaar D. de Vos W. M. Kleerebezem M. (2006). DNA micro-array-based identification of bile-responsive genes in *Lactobacillus plantarum*. J. Appl. Microbiol. 100, 728–738. 10.1111/j.1365-2672.2006.02891.x 16553727

[B8] Burns P. Sanchez B. Vinderola G. Ruas-Madiedo P. Ruiz L. Margolles A.. (2010). Inside the adaptation process of *Lactobacillus delbrueckii* subsp. lactis to bile. Int. J. Food Microbiol. 142, 132–141. 10.1016/j.ijfoodmicro.2010.06.013 20621375

[B9] Cai H. Thompson R. Budinich M. F. Broadbent J. R. Steele J. L. (2010). Genome sequence and comparative genome analysis of *Lactobacillus casei*: insights into their niche-associated evolution. Genome Biol. Evol. 1, 239–257. 10.1093/gbe/evp019 20333194 PMC2817414

[B10] Cotter P. D. Hill C. (2003). Surviving the acid test: responses of gram-positive bacteria to low pH. Microbiol. Mol. Biol. Rev. 67, 429–453. 10.1128/MMBR.67.3.429-453.2003 12966143 PMC193868

[B11] De Angelis M. Gobbetti M. (2004). Environmental stress responses in *Lactobacillus*: a review. Proteomics 4, 106–122. 10.1002/pmic.200300497 14730676

[B12] de Roos N. M. Katan M. B. (2000). Effects of probiotic bacteria on diarrhea, lipid metabolism and carcinogenesis: a review of papers published between 1988 and 1998. Am. J. Clin. Nutr. 71, 405–411. 10.1093/ajcn/71.2.405 10648252

[B13] Detmers F. J. Lanfermeijer F. C. Poolman B. (2001). Peptides and ATP binding cassette peptide transporters. Res. Microbiol. 152, 245–258. 10.1016/S0923-2508(01)01196-2 11421272

[B14] Douillard F. P. Ribbera A. Jarvinen H. M. Kant R. Pietila T. E. Randazzo C.. (2013). Comparative genomic and functional analysis of *Lactobacillus casei* and *Lactobacillus rhamnosus* strains marketed as probiotics. Appl. Environ. Microbiol. 79, 1923–1933. 10.1128/AEM.03467-12 23315726 PMC3592221

[B15] Duar R. M. Lin X. B. Zheng J. Martino M. E. Grenier T. Pérez-Muñoz M. E.. (2017). Lifestyles in transition: evolution and natural history of the genus *Lactobacillus*. FEMS Microbiol. Rev. 41, S27–S48. 10.1093/femsre/fux030 28673043

[B16] Gury J. Seraut H. Tran N. P. Barthelmebs L. Weidmann S. Gervais P.. (2009). Inactivation of PadR, the repressor of the phenolic acid stress response, by molecular interaction with Usp1, a universal stress protein from *Lactobacillus plantarum*, in *Escherichia coli*. Appl. Environ. Microbiol. 75, 5273–5283. 10.1128/AEM.00774-09 19542339 PMC2725474

[B17] Hamon E. Horvatovich P. Bisch M. Bringel F. Marchioni E. Aoudé-Werner D.. (2012). Investigation of biomarkers of bile tolerance in *Lactobacillus casei* using comparative proteomics. J. Proteome Res. 11, 109–118. 10.1021/pr200828t 22040141

[B18] Hamon E. Horvatovich P. Izquierdo E. Bringel F. Marchioni E. Aoudé-Werner D.. (2011). Comparative proteomic analysis of *Lactobacillus plantarum* for the identification of key proteins in bile tolerance. BMC Microbiol. 11:63. 10.1186/1471-2180-11-63 21447177 PMC3073879

[B19] Hannig C. Hannig M. Rehmer O. Braun G. Hellwig E. Al-Ahmad A. (2007). Fluorescence microscopic visualization and quantification of initial bacterial colonization on enamel *in situ*. Arch. Oral Biol. 52, 1048–1056. 10.1016/j.archoralbio.2007.05.006 17603998

[B20] Hosseini Nezhad M. Hussain M. A. Britz M. L. (2015). Stress Responses in Probiotic *Lactobacillus casei*. Crit. Rev. Food Sci. Nutr. 55, 740–749. 10.1080/10408398.2012.675601 24915363

[B21] Ingrassia I. Leplingard A. Darfeuille-Michaud A. (2005). *Lactobacillus casei* DN-114 001 inhibits the ability of adherent-invasive *Escherichia coli* isolated from Crohn's disease patients to adhere to and to invade intestinal epithelial cells. Appl. Environ. Microbiol. 71, 2880–2887. 10.1128/AEM.71.6.2880-2887.2005 15932981 PMC1151832

[B22] Judicial Commission of the International Committee on Systematics of Bacteria (2008). The type strain of *Lactobacillus casei* is ATCC 393, ATCC 334 cannot serve as the type because it represents a different taxon, the name *Lactobacillus paracasei* and its subspecies names are not rejected and the revival of the name “*Lactobacillus zeae”* contravenes Rules 51b (1) and (2) of the International Code of Nomenclature of Bacteria. Opinion 82. Int. J. Syst. Evol. Microbiol. 58, 1764–1765. 10.1099/ijs.0.2008/005330-0 18599731

[B23] Koskenniemi K. Laakso K. Koponen J. Kankainen M. Greco D. Auvinen P.. (2011). Proteomics and transcriptomics characterization of bile stress response in probiotic *Lactobacillus rhamnosus* GG. Mol. Cell. Proteomics 10:M110.002741. 10.1074/mcp.M110.002741 21078892 PMC3033674

[B24] Kvint K. Nachin L. Diez A. Nyström T. (2003). The bacterial universal stress protein: function and regulation. Curr. Opin. Microbiol. 6, 140–145. 10.1016/S1369-5274(03)00025-0 12732303

[B25] Licandro-Seraut H. Brinster S. van de Guchte M. Scornec H. Maguin E. Sansonetti P.. (2012). Development of an efficient *in vivo* system (Pjunc-TpaseIS1223) for random transposon mutagenesis of *Lactobacillus casei*. Appl. Environ. Microbiol. 78, 5417–5423. 10.1128/AEM.00531-12 22610425 PMC3416393

[B26] Licandro-Seraut H. Gury J. Tran N. P. Barthelmebs L. Cavin J. F. (2008). Kinetics and intensity of the expression of genes involved in the stress response tightly induced by phenolic acids in *Lactobacillus plantarum*. J. Mol. Microbiol. Biotechnol. 14, 41–47. 10.1159/000106081 17957109

[B27] Licandro-Seraut H. Scornec H. Pédron T. Cavin J.-F. Sansonetti P. J. (2014). Functional genomics of *Lactobacillus casei* establishment in the gut. Proc. Natl. Acad. Sci. U.S.A. 111, E3101–E3109. 10.1073/pnas.1411883111 25024222 PMC4121828

[B28] Livak K. J. Schmittgen T. D. (2001). Analysis of relative gene expression data using real-time quantitative PCR and the 2–ΔΔCT method. Methods 25, 402–408. 10.1006/meth.2001.1262 11846609

[B29] Munoz-Provencio D. Rodriguez-Diaz J. Collado M. C. Langella P. Bermudez-Humaran L. G. Monedero V. (2012). Functional analysis of the *Lactobacillus casei* BL23 sortases. Appl. Environ. Microbiol. 78, 8684–8693. 10.1128/AEM.02287-12 23042174 PMC3502915

[B30] Papadimitriou K. Alegría Á. Bron P. A. de Angelis M. Gobbetti M. Kleerebezem M.. (2016). Stress physiology of lactic acid bacteria. Microbiol. Mol. Biol. Rev. 80, 837–890. 10.1128/MMBR.00076-15 27466284 PMC4981675

[B31] Papadimitriou K. Zoumpopoulou G. Foligné B. Alexandraki V. Kazou M. Pot B.. (2015). Discovering probiotic microorganisms: *in vitro, in vivo*, genetic and omics approaches. Front. Microbiol. 6:58. 10.3389/fmicb.2015.00058 25741323 PMC4330916

[B32] Perpetuini G. Pham-Hoang B. N. Scornec H. Tofalo R. Schirone M. Suzzi G.. (2016). In *Lactobacillus pentosus*, the olive brine adaptation genes are required for biofilm formation. Int. J. Food Microbiol. 216, 104–109. 10.1016/j.ijfoodmicro.2015.10.002 26447789

[B33] Perpetuini G. Scornec H. Tofalo R. Serror P. Schirone M. Suzzi G.. (2013). Identification of critical genes for growth in olive brine by transposon mutagenesis of *Lactobacillus pentosus* C11. Appl. Environ. Microbiol. 79, 4568–4575. 10.1128/AEM.01159-13 23686273 PMC3719503

[B34] Price M. N. Deutschbauer A. M. Skerker J. M. Wetmore K. M. Ruths T. Mar J. S.. (2013). Indirect and suboptimal control of gene expression is widespread in bacteria. Mol. Syst. Biol. 9:660. 10.1038/msb.2013.16 23591776 PMC3658271

[B35] Saxelin M. Tynkkynen S. Mattila-Sandholm T. de Vos W. M. (2005). Probiotic and other functional microbes: from markets to mechanisms. Curr. Opin. Biotechnol. 16, 204–211. 10.1016/j.copbio.2005.02.003 15831388

[B36] Scornec H. Tichit M. Bouchier C. Pédron T. Cavin J.-F. Sansonetti P. J.. (2014). Rapid 96-well plates DNA extraction and sequencing procedures to identify genome-wide transposon insertion sites in a difficult to lyse bacterium: *Lactobacillus casei*. J. Microbiol. Methods 106, 78–82. 10.1016/j.mimet.2014.08.001 25135488

[B37] Smokvina T. Wels M. Polka J. Chervaux C. Brisse S. Boekhorst J.. (2013). *Lactobacillus paracasei* comparative genomics: towards species pan-genome definition and exploitation of diversity. PLoS ONE 8:e68731. 10.1371/journal.pone.0068731 23894338 PMC3716772

[B38] Stevens M. J. A. Molenaar D. de Jong A. de Vos W. M. Kleerebezem M. (2010). Involvement of the mannose phosphotransferase system of *Lactobacillus plantarum* WCFS1 in peroxide stress tolerance. Appl. Environ. Microbiol. 76, 3748–3752. 10.1128/AEM.00073-10 20363783 PMC2876449

[B39] Sugimoto S. Abdullah-Al-Mahin Sonomoto K. (2008). Molecular chaperones in lactic acid bacteria: physiological consequences and biochemical properties. J. Biosci. Bioeng. 106, 324–336. 10.1263/jbb.106.324 19000607

[B40] Tawakoli P. N. Al-Ahmad A. Hoth-Hannig W. Hannig M. Hannig C. (2013). Comparison of different live/dead stainings for detection and quantification of adherent microorganisms in the initial oral biofilm. Clin. Oral Invest. 17, 841–850. 10.1007/s00784-012-0792-3 22821430

[B41] Tong Y. Zhai Q. Lu W. Tian F. Zhao J. Zhang H.. (2017). New insights in integrated response mechanism of *Lactobacillus plantarum* under excessive manganese stress. Food Res. Int. 102, 323–332. 10.1016/j.foodres.2017.10.014 29195955

[B42] Tran N. P. Gury J. Dartois V. Nguyen T. K. C. Seraut H. Barthelmebs L.. (2008). Phenolic acid-mediated regulation of the padC gene, encoding the phenolic acid decarboxylase of *Bacillus subtilis*. J. Bacteriol. 190, 3213–3224. 10.1128/JB.01936-07 18326577 PMC2347383

[B43] Untergasser A. Nijveen H. Rao X. Bisseling T. Geurts R. Leunissen J. A. M. (2007). Primer3Plus, an enhanced web interface to Primer3. Nucleic Acids Res. 35, W71–W74. 10.1093/nar/gkm306 17485472 PMC1933133

[B44] van de Guchte M. Serror P. Chervaux C. Smokvina T. Ehrlich S. D. Maguin E. (2002). Stress responses in lactic acid bacteria. Antonie Van Leeuwenhoek 82, 187–216. 10.1023/A:1020631532202 12369188

[B45] Wang G. Li D. Ma X. An H. Zhai Z. Ren F.. (2015). Functional role of oppA encoding an oligopeptide-binding protein from *Lactobacillus salivarius* Ren in bile tolerance. J. Ind. Microbiol. Biotechnol. 42, 1167–1174. 10.1007/s10295-015-1634-5 25998246

[B46] Wu C. Zhang J. Chen W. Wang M. Du G. Chen J. (2012). A combined physiological and proteomic approach to reveal lactic-acid-induced alterations in *Lactobacillus casei* Zhang and its mutant with enhanced lactic acid tolerance. Appl. Microbiol. Biotechnol. 93, 707–722. 10.1007/s00253-011-3757-6 22159611

[B47] Wu R. Sun Z. Wu J. Meng H. Zhang H. (2010). Effect of bile salts stress on protein synthesis of *Lactobacillus casei* Zhang revealed by 2-dimensional gel electrophoresis. J. Dairy Sci. 93, 3858–3868. 10.3168/jds.2009-2967 20655455

[B48] Xie Y. Chou L. Cutler A. Weimer B. (2004). DNA Macroarray profiling of *Lactococcus lactis* subsp. lactis IL1403 gene expression during environmental stresses. Appl. Environ. Microbiol. 70, 6738–6747. 10.1128/AEM.70.11.6738-6747.2004 15528540 PMC525116

[B49] Yamasaki-Yashiki S. Sawada H. Kino-oka M. Katakura Y. (2017). Analysis of gene expression profiles of *Lactobacillus paracasei* induced by direct contact with *Saccharomyces cerevisiae* through recognition of yeast mannan. Biosci. Microbiota Food Health 36, 17–25. 10.12938/bmfh.BMFH-2016-015 28243547 PMC5301053

[B50] Zotta T. Guidone A. Tremonte P. Parente E. Ricciardi A. (2012). A comparison of fluorescent stains for the assessment of viability and metabolic activity of lactic acid bacteria. World J. Microbiol. Biotechnol. 28, 919–927. 10.1007/s11274-011-0889-x 22805812

